# The association of healthy behaviors with cognitive health expectancy in Chinese older adults: a population-based cohort study

**DOI:** 10.18632/aging.103617

**Published:** 2020-09-09

**Authors:** Chengbei Hou, Yinan Lin, Zachary Zimmer, Xianghua Fang

**Affiliations:** 1 Center for Evidence-Based Medicine, Xuanwu Hospital, Capital Medical University, Beijing, China; 2 Center for Applied Statistics and School of Statistics, Renmin University of China, Beijing, China; 3 Department of Family Studies and Gerontology, Mount Saint Vincent University, Halifax, Nova Scotia, Canada

**Keywords:** cognitive impairment, life expectancy, life styles, leisure activities, Markov multistate model

## Abstract

Objective: To examine how lifestyles and leisure activities are associated with cognitive health expectancy among older adults.

Results: For young-old (aged 65), an absolute increase in life years without cognitive impairment was found among those with a healthy diet, engaging in mental activities and in social activities. For old-old (aged 85), an absolute increase was found for men engaging in physical activities besides those. Compared with counterparts in a high risk group, the young-old in a medium-low risk group had a smaller proportion of years without cognitive impairment. Old-old in a low risk group had a greater proportion.

Conclusion: Extra years of life gained by a healthy dietary pattern, mental activities, and social activities are free of cognitive impairment for both sexes across ages. The beneficial impact of individual and combined modifiable factors on cognitive health is most prominent in old-old.

Methods: Data come from The Chinese Longitudinal Healthy Longevity Survey, a population-based cohort study of 27,193 participants aged 65+ conducted between 2002 and 2014. Smoking status, alcohol consumption, dietary pattern, marital status, physical, mental, social, and productive activities were assessed at baseline. Cognitive status was measured using the Chinese version of the MMSE.

## INTRODUCTION

Cognitive impairment (CI) is prevalent in older adults and imposes burdens on families and health care systems [1]. Reducing the risk of developing CI takes on added importance with the absence of disease-modifying treatment. In addition, nearly every region and country in the world is experiencing rapid population aging [2]. The general consensus is that longevity is a multifactorial quantitative trait that is influenced by biological, environmental, and psychosocial characteristics [3]. The link between age and CI suggests that increasing longevity and population aging can result in a higher CI burden. Thus, modifiable factors which have the capacity to both prevent cognitive decline and prolong life among older persons are particularly important given their amenability to intervention.

A broad range of prospective cohort studies have suggested that healthy lifestyles and leisure activities are modifiable factors that decrease the risk of CI in old age [4, 5]. Other research indicates these factors also serve to reduce mortality [6]. What previous research has not determined however is the extent to which these modifiable factors affect total life expectancy and years of life without CI. It is the concurrent influence of these factors on prolonging total and cognitive healthy life that is important for promoting healthy aging. One way of assessing these concurrent effects is by calculating health expectancies, which combine information on morbidity and mortality in order estimate length of life and partition total life into different health states [7].

Using health expectancy methods, the current study investigated the association of several modifiable factors including lifestyle (i.e., smoking status, alcohol intake, and dietary pattern), marital status, and leisure (i.e., physical activities, mental activities, social activities, and productive activities), examined separately and in combination, with total and cognitive impairment-free life expectancy.

## RESULTS

### Characteristics of participants at baseline

The sample contained 27,193 individuals (15,800 women). 45.6% of the sample was observed across two waves, 30.2% across three waves, 15.4% across four waves and 8.8% across five waves. That is, all participants were observed for a minimum of two waves, including a baseline and at least one follow-up. At baseline 22.1% of men and 44.9% of women had CI (Table 1). The median time from baseline to follow-up was 5.1 years. During the observation period, 4,161 participants experienced onset of CI, and 18,711 died. For both sexes, the CI-free group was younger than the CI group (men: mean age, 82.3 years vs 93.4 years; women: mean age, 84.7 years vs 96.6 years); a greater proportion of CI-free individuals in comparison to the impaired received any formal education, lived in cities, engaged in white-collar work, had no functional limitation, had a healthy dietary pattern, were married, and participated leisure activities.

**Table 1:** Characteristics of study population at baseline by cognitive status for men and women.

**Variables**	**Men**			**Women**
	**Free of CI**	**With CI**		**Free of CI**	**With CI**
No. (%)	8870 (77.9)	2523 (22.1)		8689 (55.1)	7111 (44.9)
**Sociodemographic characteristics**					
Mean (SD) age	82.3 (10.6)	93.4 (7.5)		84.7 (11.8)	96.6 (7.0)
Region of residence (missing=0)					
Rural	5190 (45.6)	1639 (14.3)		5095 (32.3)	4636 (29.3)
Urban	3680 (32.3)	884 (7.8)		3594 (22.7)	2475 (15.7)
Educational attainment (missing=125)					
No formal education (0y)	2878 (25.4)	1422 (12.5)		6686 (42.5)	6543 (41.6)
Primary school (1-6y)	4303 (37.9)	896 (7.9)		1547 (9.8)	454 (2.9)
Middle school or higher (7+y)	1671 (14.7)	183 (1.6)		421 (2.8)	64 (0.4)
Primary lifetime occupation (missing=72)					
White collar	1295 (11.4)	159 (1.4)		319 (2.0)	68 (0.4)
Others	7560 (66.5)	2357 (20.7)		8343 (53.0)	7020 (44.6)
Economic condition (missing=132)					
Good	1633 (14.4)	306 (2.7)		1430 (9.1)	833 (5.3)
Fair	5989 (52.8)	1623 (14.3)		5832 (37.1)	4637 (29.5)
Poor	1202 (10.6)	590 (5.2)		1383 (8.8)	1603 (10.2)
**Health status**					
Functional limitation (missing=101)					
No	7865 (69.2)	1299 (11.5)		7018 (44.6)	2943 (18.7)
Yes	985 (8.7)	1208 (10.6)		1641 (10.4)	4133 (26.3)
Number of chronic diseases^a^ (missing=27)				
0	4993 (43.8)	1483 (13.1)		4739 (30.0)	4385 (27.8)
1	2606 (22.9)	717 (6.3)		2636 (16.7)	1907 (12.1)
≥2	1266 (11.1)	322 (2.8)		1302 (8.3)	810 (5.1)
**Modifiable factors**					
Smoking status (missing=47)					
Never smoking	3403 (29.9)	1154 (10.1)		7486 (47.5)	6197 (39.3)
Former smoking	2211 (19.4)	663 (5.8)		539 (3.4)	498 (3.2)
Current smoking	3248 (28.5)	698 (6.1)		649 (4.1)	400 (2.5)
Alcohol intake (missing=28)					
Drinking	3078 (27.1)	601 (5.3)		928 (5.9)	809 (5.1)
No drinking	5786 (50.8)	1918 (16.8)		7754 (49.1)	6291 (39.9)
Dietary pattern (missing=18)					
Healthy	3390 (29.8)	623 (5.5)		3236 (20.5)	2010 (12.7)
Unhealthy	5471 (48.1)	1900 (16.6)		5451 (34.5)	5094 (32.3)
Marital status (missing=3)					
In marriage	4620 (40.6)	640 (5.6)		2168 (13.7)	301 (1.9)
Not in marriage	4248 (37.3)	1883 (16.5)		6521 (41.3)	6809 (43.1)
Physical activities (missing=44)					
Yes	3589 (31.6)	509 (4.5)		2514 (15.9)	909 (5.8)
No	5267 (46.3)	2009 (17.6)		6163 (39.1)	6189 (39.2)
Mental activities (missing=0)					
Yes	2674 (23.5)	171 (1.5)		645 (4.1)	57 (0.4)
No	6196 (54.4)	2352 (20.6)		8044 (50.9)	7054 (44.6)
Social activities (missing=2)					
Yes	1873 (16.4)	134 (1.2)		1197 (7.6)	166 (1.1)
No	6995 (61.4)	2389 (21.0)		7492 (47.3)	6945 (44.0)
Productive activities (missing=1)					
Yes	5563 (48.8)	598 (5.2)		5924 (37.5)	1673 (10.6)
**No**	**3306 (29.0)**	**1925 (16.9)**		**2765 (17.5)**	**5438 (34.4)**

CI= cognitive impairment.

^a^ Included hypertension, diabetes mellitus, cardiovascular disease, stroke, respiratory disease, and cancer.

### Association between independent modifiable factor and cognitive health expectancy

Supplementary Figure 1 shows the total and cognitive impaired life expectancies by characteristics of each modifiable factor after adjustment. Participants characterized with more salutary behaviors, including never smoking, not drinking, eating a healthy diet, being married, and engaging in physical, mental, social, and productive activities, lived longer than counterparts without these factors across ages. The life expectancy advantage ranged from 0.7 (0.3 to 1.1) to 3.4 (2.3 to 3.9) years for individuals aged 65 and from 0.3 (0.1 to 0.5) to 1.8 (0.9 to 2.2) years for those aged 85. Note that in comparison to current smokers, former smokers had slightly shorter lifespan.

Table 2 presents estimates for total and cognitive impairment-free life expectancy, and the proportion of life free of CI, by modifiable factors after adjustment, for those aged 65. There was an absolute increase in life years without CI for those with a healthy dietary pattern, married men, and those that undertake mental and social activities. To provide a concrete example, a 65 year old man with a healthy dietary pattern was estimated to live 12.8 years in total, with 12.1 of these being without CI. His counterpart with an unhealthy pattern was expected to live 12.0 total years of which 11.1 were without CI. Therefore, the healthy dietary pattern resulted in a 0.8 (0.3 to 1.3) year advantage in total life and a 1.0 (0.5 to 1.5) year advantage in life expected without CI. Therefore, we can conclude that a healthy dietary pattern is associated with a longer life that is free of CI.

**Table 2:** Differences in total life expectancy and cognitive impairment-free life expectancy, and proportion of life free of cognitive impairment by modifiable factors for men and women at age 65 years.

**Variables^a^**	**TLE**	**CIFLE**	**Proportion of CIFLE (%)**	**Difference in TLE (95%CI)**	**Difference in CIFLE (95%CI)**	**Difference in proportion of CIFLE (95%CI)**
**Men**						
Smoking status						
Current	10.9	9.9	90.8	Reference	Reference	Reference
Never	11.8	10.6	89.5	1.0 (0.5 to 1.4)	0.7 (0.3 to 1.1)	-1.3 (-2.4 to -0.4)
Alcohol intake						
No drinking	12.1	11.3	93.4	Reference	Reference	Reference
Drinking	12.8	11.9	93.1	0.7 (0.3 to 1.2)	0.6 (0.3 to 1.1)	0.2 (-0.5 to 0.6)
Dietary pattern						
Unhealthy	12.0	11.1	92.6	Reference	Reference	Reference
Healthy	12.8	12.1	94.4	0.8 (0.3 to 1.3)	1.0 (0.5 to 1.5)	1.8 (1.1 to 3.1)
Marital status						
Not in marriage	11.3	10.3	91.0	Reference	Reference	Reference
In marriage	13.2	12.4	94.0	1.9 (1 to 2.4)	2.1 (1.3 to 2.7)	3.0 (1.9 to 5.9)
Physical activities						
No	12.0	11.2	93.2	Reference	Reference	Reference
Yes	12.8	12.0	93.6	0.8 (0.3 to 1.2)	0.8 (0.3 to 1.2)	0.4 (-0.2 to 1.4)
Mental activities						
No	11.7	10.8	92.6	Reference	Reference	Reference
Yes	13.1	12.4	94.4	1.5 (0.8 to 2.2)	1.6 (0.9 to 2.2)	1.8 (0.8 to 3.6)
Social activities						
No	11.5	10.7	93.0	Reference	Reference	Reference
Yes	13.3	12.6	94.3	1.8 (1.1 to 2.4)	1.9 (1.2 to 2.4)	1.3 (0.6 to 2.7)
Productive activities						
No	10.9	10.2	93.1	Reference	Reference	Reference
Yes	13.8	12.9	93.5	2.9 (2.0 to 3.4)	2.7 (2.0 to 3.3)	0.4 (-0.2 to 1.3)
**Women**						
Smoking status						
Current	12.8	11.1	86.6	Reference	Reference	Reference
Never	13.8	11.8	85.4	1.0 (0.5 to 1.4)	0.7 (0.3 to 1.1)	-1.2 (-2.4to -0.1)
Alcohol intake						
No drinking	14.1	12.7	89.9	Reference	Reference	Reference
Drinking	15.0	13.5	90.5	0.9 (0.4 to 1.3)	0.8 (0.4 to 1.3)	0.6 (-0.5 to 1.6)
Dietary pattern						
Unhealthy	14.0	12.3	88.3	Reference	Reference	Reference
Healthy	15.0	13.7	91.5	1.0 (0.4 to 1.6)	1.4 (0.9 to 1.9)	3.2 (2.1 to 4.9)
Marital status						
Not in marriage	13.8	12.4	90.3	Reference	Reference	Reference
In marriage	15.3	13.7	89.6	1.5 (0.9 to 2.0)	1.3 (0.7 to 1.7)	-0.7 (-1.8 to 0.2)
Physical activities						
No	14.1	12.7	90.3	Reference	Reference	Reference
Yes	14.8	13.2	89.0	0.7 (0.3 to 1.1)	0.5 (0.1 to 0.8)	-1.3 (-2.4 to -0.6)
Mental activities						
No	13.7	12.2	89.0	Reference	Reference	Reference
Yes	15.4	14.0	91.3	1.6 (1.0 to 2.3)	1.8 (1.1 to 2.5)	2.3 (0.8 to 4.2)
Social activities						
No	13.7	12.1	88.5	Reference	Reference	Reference
Yes	15.4	13.9	90.2	1.7 (1.2 to 2.4)	1.8 (1.2 to 2.3)	1.7 (0.7 to 3.1)
Productive activities						
No	12.7	11.1	87.5	Reference	Reference	Reference
Yes	16.1	14.4	89.7	3.4 (2.3 to 3.9)	3.3 (2.5 to 3.8)	2.2 (1.3 to 4.6)

TLE= total life expectancy; CIFLE= cognitive impairment-free life expectancy.

^a^ Adjusted for age, region of residence, educational attainment, primary lifetime occupation, economic condition, functional limitation, chronic diseases, and variables included in the table.

There was a relative increase in life years without CI for women engaging in productive activities. Those engaging in productive activities were expected to live 14.4 years without CI compared to only 11.1 for those not engaging in a productive activities, a 3.3 (2.5 to 3.8) year advantage. Although this advantage was less than the 3.4 (2.3 to 3.9) year advantage in total life, the proportion of years lived free of CI increased significantly by 2.2 (1.3 to 4.6) percentage points for women that engage in productive activities.

For never smoking and physical activity among women, we observed a relative reduction in life years without CI. For instance, compared with those not engaging in physical activity, women engaging in physical activity gained 0.5 (0.1 to 0.8) year of life without CI, but the proportion of years lived free of CI was reduced significantly by 1.3 (0.6 to 2.4) percentage points.

Table 3 presents estimates for total and cognitive impairment-free life expectancy, and the proportion of life free of CI for those aged 85. We found an absolute increase in life years without CI for those with a healthy dietary pattern, men engaging in physical activities, and those engaging in mental and social activities. There was a relative increase in life years without CI for women never smoking, those married, women engaging in physical activities, and those engaging in productive activities.

**Table 3:** Differences in total life expectancy and cognitive impairment-free life expectancy, and proportion of life free of cognitive impairment by modifiable factors for men and women at age 85 years.

**Variables^a^**	**TLE**	**CIFLE**	**Proportion of CIFLE (%)**	**Difference in TLE (95%CI)**	**Difference in CIFLE (95%CI)**	**Difference in proportion of CIFLE (95%CI)**
**Men**						
Smoking status						
Current	3.3	2.5	74.9	Reference	Reference	Reference
Never	3.7	2.8	76.1	0.4 (0.3 to 0.6)	0.3 (0.2 to 0.5)	1.2 (-0.7 to 3.3)
Alcohol intake						
No drinking	3.7	3.0	80.6	Reference	Reference	Reference
Drinking	4.0	3.3	80.5	0.3 (0.1 to 0.5)	0.2 (0.1 to 0.4)	-0.1 (-1.6 to 1.6)
Dietary pattern						
Unhealthy	3.7	2.9	77.8	Reference	Reference	Reference
Healthy	4.1	3.5	84.9	0.4 (0.1 to 0.7)	0.6 (0.3 to 0.9)	7.1 (4.7 to 11.8)
Marital status						
Not in marriage	3.5	2.8	78.8	Reference	Reference	Reference
In marriage	4.3	3.5	81.9	0.8 (0.3 to 1.0)	0.7 (0.3 to 1.0)	3.1 (1.4 to 6.4)
Physical activities						
No	3.6	2.8	78.3	Reference	Reference	Reference
Yes	4.2	3.5	84.1	0.6 (0.1 to 0.9)	0.7 (0.4 to 0.9)	5.8 (3.5 to 11.8)
Mental activities						
No	3.6	2.8	77.8	Reference	Reference	Reference
Yes	4.3	3.7	86.1	0.8 (0.3 to 1.2)	1.0 (0.5 to 1.4)	8.3 (4.7 to 13.9)
Social activities						
No	3.5	2.7	77.7	Reference	Reference	Reference
Yes	4.5	3.9	87.6	1.0 (0.4 to 1.5)	1.2 (0.7 to 1.6)	9.9 (6.4 to 18.0)
Productive activities						
No	3.2	2.5	76.6	Reference	Reference	Reference
Yes	4.6	3.8	82.8	1.4 (0.7 to 1.9)	1.3 (0.8 to 1.8)	6.2 (3.7 to 12.0)
**Women**						
Smoking status						
Current	4.1	2.4	59.4	Reference	Reference	Reference
Never	4.5	2.8	63.1	0.4 (0.3 to 0.6)	0.4 (0.3 to 0.6)	3.7 (1.4 to 6.3)
Alcohol intake						
No drinking	4.5	3.1	68.5	Reference	Reference	Reference
Drinking	4.9	3.4	69.3	0.4 (0.2 to 0.6)	0.3 (0.1 to 0.5)	0.8 (-1.2 to 2.9)
Dietary pattern						
Unhealthy	4.5	2.9	65.2	Reference	Reference	Reference
Healthy	5.0	3.7	73.7	0.5 (0.1 to 0.8)	0.8 (0.4 to 1.0)	8.5 (6.3 to 11.3)
Marital status						
Not in marriage	4.3	2.9	66.7	Reference	Reference	Reference
In marriage	5.2	3.7	71.9	0.9 (0.4 to 1.2)	0.8 (0.5 to 1.1)	5.2 (3.1 to 8.4)
Physical activities						
No	4.4	3.0	67.7	Reference	Reference	Reference
Yes	5.1	3.6	70.6	0.7 (0.3 to 0.8)	0.6 (0.3 to 0.8)	2.9 (0.9 to 5.5)
Mental activities						
No	4.4	2.9	66.5	Reference	Reference	Reference
Yes	5.5	4.6	82.4	1.2 (0.3 to 1.8)	1.6 (1.0 to 2.2)	15.9 (10.7 to 24.4)
Social activities						
No	4.3	2.9	66.6	Reference	Reference	Reference
Yes	5.3	3.9	74.4	1.0 (0.5 to 1.4)	1.1 (0.7 to 1.4)	7.8 (4.8 to 12.3)
Productive activities						
No	3.8	2.3	60.2	Reference	Reference	Reference
Yes	5.6	3.9	70.6	1.8 (0.9 to 2.2)	1.6 (1.0 to 2.0)	10.4 (7.5 to 15.3)

TLE= total life expectancy; CIFLE= cognitive impairment-free life expectancy.

^a^ Adjusted for age, region of residence, educational attainment, primary lifetime occupation, economic condition, functional limitation, chronic diseases, and variables included in the table.

### Association between combined modifiable factors and cognitive health expectancy

**Figure 1:**
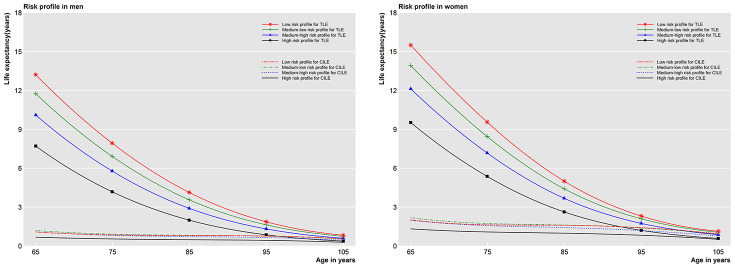
**Total life expectancy and cognitive impaired life expectancy in four risk profiles after adjustment.** TLE=Total life expectancy; CILE= Cognitive impaired life expectancy.

When combining modifiable factors into risk profiles, those with a lower risk profile had longer total life expectancy (Figure 1). Table 4 provides the results across risk profiles for men and women aged 65 and 85. For people aged 65, individuals in the high risk profile group had a 1.4 (0.3 to 2.4) and 1.8 (0.4 to 3.3) percentage points increase in the proportion of years without CI for men and women, respectively, over those in the medium-low risk profile group. However, for those aged 85, individuals in the high risk profile group had a 4.6 (0.1 to 8.8) and a 5.6 (0 to 11.0) percentage points decrease in proportion of years without CI for men and women, respectively, over their counterparts in the low risk profile group.

**Table 4:** Differences in total life expectancy and cognitive impairment-free life expectancy, and proportion of life free of cognitive impairment by four risk profile groups for men and women at age 65 and 85 years.

**Age**	**Variables^a^**	**High risk profile**	**Medium-high risk profile**	**Medium-low risk profile**	**Low risk profile**	**Difference of Medium-high *vs* High (95%CI)**	**Difference of Medium-low *vs* High (95%CI)**	**Difference of low *vs* High (95%CI)**
65y	Men							
	TLE	7.7	10.1	11.8	13.2	2.4 (2.0 to 2.9)	4.0 (3.4 to 4.6)	5.5 (4.6 to 6.5)
	CIFLE	7.0	9.0	10.6	12.1	2.0 (1.7 to 2.5)	3.5 (3.1 to 4.1)	5.1 (4.3 to 6.2)
	Proportion (%)	91.2	89.2	89.8	91.8	-2.0 (-2.7 to -1.1)	-1.4 (-2.4 to -0.3)	0.6 (-1.1 to 2.4)
	Women							
	TLE	9.5	12.1	13.9	15.5	2.6 (2.2 to 3.1)	4.4 (3.8 to 4.9)	6.0 (5.1 to 6.9)
	CIFLE	8.2	10.1	11.7	13.5	1.9 (1.6 to 2.5)	3.5 (3.1 to 4.2)	5.3 (4.7 to 6.4)
	Proportion (%)	86.1	83.7	84.3	87.0	-2.4 (-3.7 to -1.2)	-1.8 (-3.3 to -0.4)	0.9 (-1.8 to 3.4)
85y	Men							
	TLE	2.0	2.9	3.6	4.1	0.9 (0.7 to 1.1)	1.6 (1.3 to 1.8)	2.1 (1.8 to 2.6)
	CIFLE	1.5	2.2	2.7	3.3	0.7 (0.5 to 0.8)	1.2 (1.0 to 1.4)	1.8 (1.4 to 2.2)
	Proportion (%)	75.8	75.1	76.7	80.4	-0.7 (-2.2 to 1.0)	0.9 (-1.2 to 3.0)	4.6 (0.1 to 8.8)
	Women							
	TLE	2.6	3.7	4.4	5.0	1.0 (0.9 to 1.2)	1.8 (1.5 to 2.0)	2.4 (2.0 to 2.9)
	CIFLE	1.6	2.2	2.8	3.4	0.6 (0.5 to 0.8)	1.1 (0.9 to 1.3)	1.8 (1.5 to 2.1)
	Proportion (%)	62.4	61.3	63.3	68.0	-1.1 (-3.0 to 1.1)	0.9 (-1.7 to 3.5)	5.6 (0 to 11.0)

TLE= total life expectancy; CIFLE= cognitive-impairment-free life expectancy.

^a^ Adjusted for age, region of residence, educational attainment, primary lifetime occupation, economic condition, functional limitation, and chronic diseases.

Sensitivity analyses indicated similar results when not adjusting for potential mediators, with a few exceptions. Social activity did not increase or decrease percent of life without CI for women aged 65 (Supplementary Tables 1 and 2). After excluding onset of CI events during the first follow-up and deaths in the first year, more modifiable factors resulted in an absolute increase in life years without CI in comparison to the main analysis, especially for the oldest old (added factors: physical activities among men aged 65, never smoking among women aged 85, and productive activities for all participants aged 85) (Supplementary Tables 3 and 4). None of the sensitivity tests affected the analyses of combined modifiable factors (Supplementary Tables 5 and 6).

## DISCUSSION

Findings from this nationwide study of older adults in China demonstrated that healthy behaviors expanded total years of life, congruent with prior research [8, 9]. However, whether this gain in total life expectancy was made up of years with or without CI depended upon specific factors.

Two opposing processes appear to be at work. On one hand, healthy behaviors may postpone incidence of CI and/or reduce mortality among those without CI, both of which increase years lived free of CI. On the other hand, some factors may lower the risk of death among those with CI, which results in more years with CI [10]. It is worth noting that the extra years of life gained by a healthy diet, mental and social activities are free of CI for both sexes across young-old and old-old in our study. Moreover, compared with the young-old, these modifiable factors had greater impact on cognitive health for the old-old adults where most of these factors increase life free of CI in either absolute or relative terms

Our evidence regarding healthy diets correspond with earlier evidence from a large number of epidemiological studies that indicate healthy dietary patterns associate with less cognitive decline and a reduced risk of mild cognitive impairment and dementia [11]. Fruits, vegetables, and legumes which are rich in antioxidants such as vitamin C, vitamin E, and flavonoids reduce oxidative stress and down-regulate neuroinflammation both of which are associated with an increased risk of CI [11, 12]. Moreover, consuming foods high in Omega-3 polyunsaturated fatty acids like fish may maintain neuronal membrane integrity and neuronal function [13].

The absolute increase in life years without CI for those engaging in mental and social activities may be attributable to the notion of cognitive reserve [14]. These activities likely increase efficiency of neural networks or result in using alternate networks more effectively after neurologic insult, delaying onset of dementia, thereby extending years of life free of CI. However, progression of the dementia-related brain pathology itself probably does not differ as a function of cognitive reserve [15]. Once a critical threshold of the pathologic severity is reached, there is no longer a role to play for cognitive reserve, and progression of dementia in terms of effects on cognitive decline is more rapid, leading to fewer CI years before death [16].

One surprising result in our study was that current smokers had a slight longevity advantage compared to former smokers. A similar result was found in a report from the Beijing Longitudinal Study of Aging (BLSA) [17]. In BLSA, researchers found that male current smokers had a longer life expectancy than corresponding short-term quitters (<5 years); female current lighter smokers lived longer compared with female ex-smokers. Since our models adjusted for several diseases related to smoking, such counterintuitive findings may be explained by survival selection [18]. Specifically, “weak smokers” may be selectively eliminated from a surviving population of smokers before age 65. In contrast, smokers with relatively hardier characteristics (e.g., unobserved genetic characteristics) are more likely to survive relative to ex-smokers.

The current study also investigated the combined effects of modifiable factors on cognitive health expectancy by constructing risk profiles. Compared to those with a high risk profile, people in lower risk profile groups live longer. For individuals aged 65, our results concur with a report from the Australian Longitudinal Studies (DYNOPTA) that showed people aged 65 without harmful behaviors (smoking, obesity, sedentary behavior) live a lesser proportion of life without CI owing to a longer total life expectancy than those with all three harmful behaviors [19]. The findings of these two studies suggest that increased longevity is a strong competing risk for CI. However, the situation is different for the old-old aged 85. For these people, the low risk profile increased the proportion of life years free of CI compared with the high risk profile group. This means the positive impact of modifiable factors on cognitive health may surpass the negative impact of ageing on cognitive health at very old ages.

### Strengths and limitations of the study

The major strengths of our study include a prospective design, a large nationwide sample, long-term follow-up, and a diversity of modifiable factors. Most importantly, previous estimates of the effect of modifiable factors on CI and death have been limited to relative risks without considering the combination of information on both quantity and quality of life. The health expectancy approach we adopt in this study provides an intuitive understanding of risk in terms of actual years of life. This is the type of understanding that can be easily communicated between doctors and patients [20].

This study also had several limitations. MMSE was used to characterize the cognitive states. Clinical assessments can provide much more accurate diagnoses of dementia, although such information is not available in population-level studies. Therefore, the MMSE was not used to determine clinical diagnosis, but as an indication of the level of MMSE impairment as suggestive of dementia. Additionally, since the CLHLS was designed to interview all voluntarily participating centenarians, there might be a potential healthy volunteer bias. However, the prevalence of CI in our study is similar to a recent estimate of oldest-old based on a meta-analysis [21]. Hence, a healthy volunteer bias on life expectancy is likely not a major problem in our study. Furthermore, the categories used to characterize some factors in our study are somewhat crude due to lack of more subtle details in the data. Therefore, some results should be interpreted with caution. For example, we found that alcohol consumption prolonged total and cognitive impairment-free life expectancy. A number of studies have shown that the risk of mortality and incident CI depended on the dose and frequency of alcohol intake, a factor we are unable to take into account given the available data [6, 22]. Further studies are needed to examine these associations using more precise measurement. Finally, results of this study are generalizable to older Chinese. Although we have adjusted our results for a series of potential confounders, confirmation in other populations is required.

In conclusion, past research has shown that certain modifiable behaviors have robust influences on life expectancy. In this study we demonstrate that the extra years of life gained by a healthy dietary pattern, mental activities, and social activities are completely free of cognitive impairment for both sexes across ages. Therefore, attention to these three factors are critical for promoting healthy longevity and they may be the most efficacious factors for reducing healthcare costs. Furthermore, the favorable impact of individual and combined modifiable factors on cognitive health is more prominent in the oldest-old in comparison to younger-old. Therefore, to achieve successful cognitive ageing, it is never too late to start a healthy lifestyle.

## MATERIALS AND METHODS

### Design and participants

Data was sourced from a cohort of elderly people who participated in the Chinese Longitudinal Healthy Longevity Survey (CLHLS), a nationwide population-based survey conducted in 22 provinces of China. The first wave was carried out in 1998, and six follow-ups with participant replacement accounting for attrition were conducted in 2000, 2002, 2005, 2008, 2011 and 2014. The first and second waves were limited to persons aged 80 and over. Those aged 65 to 79 were added in 2002 and subsequent waves. The CLHLS attempted to interview all centenarians within sampled areas and adopted a targeted random-sample design to ensure representativeness with approximately equal numbers of male and female nonagenarians, octogenarians, and young-old (aged 65-79 years) living near the centenarians. The study design details and data quality have been described elsewhere [23]. The CLHLS was approved by the ethics committee of Peking University, and written informed consent was obtained from every participant or proxy (next of kin or guardian).

**Figure 2:**
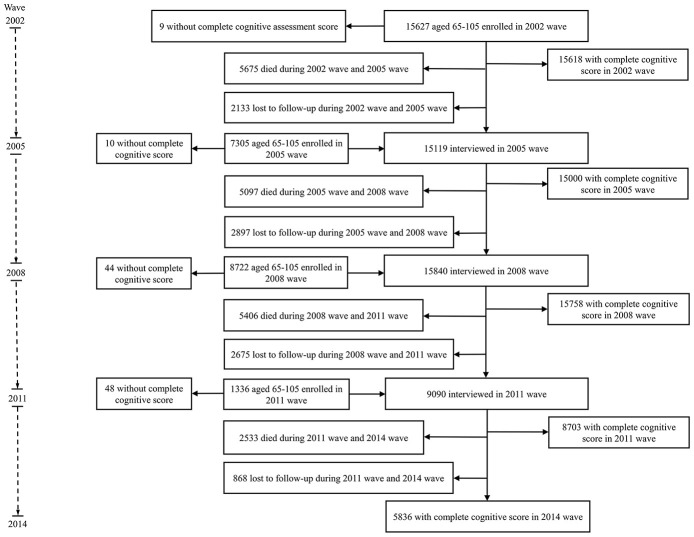
**Flow diagram of participants inclusion and follow-up.**

Because our aim is to examine health expectancy in people aged 65 and over, we draw on data from waves 2002-2014. In the 2002 wave, 15,627 people aged 65-105 were interviewed, and 7,305, 8,722 and 1,336 new participants were added in 2005, 2008 and 2011, respectively. We excluded those without any follow-up information on cognitive state or death (n=5,686) and those without information on cognitive state at baseline (n= 111). The analytical sample is 27,193 (Figure 2).

### Assessment of cognitive impairment

Global cognitive functioning was assessed by a Chinese version of the Mini-Mental State Examination (cMMSE) adapted from the international MMSE to meet the Chinese cultural and socioeconomic context [24]. cMMSE consists of 24 items covering six aspects of cognitive functioning (orientation, registration, attention, calculation, recall, and language) [25]. The maximum score is 30, with higher scores representing better functioning. “Unable to answer” was categorized as an incorrect response [26]. Cognitive status was considered as missing when the cMMSE was not completed. The MMSE was not used as a clinical diagnosis, but rather as suggestive of dementia. We used education-based MMSE cut-off points widely accepted in China as a proxy for dementia status: cMMSE score≤17=‘probable dementia’, cMMSE score≥18=‘no cognitive impairment’ for participants with no formal education, cut point of 20/21 for those with 1–6 years of education, and cut point of 24/25 for those with more than six years of education [27].

### Ascertaining death

If a subject died during the inter-survey period, the date of death was obtained through various sources including death certificates, next of kin, and neighborhood committees. All dates were validated, and those reported on death certificates were ultimately used when available; otherwise the next of kin’s report was used, followed by neighborhood registries. Mortality data in the CLHLS has been shown to be high quality [28].

### Modifiable factors

Data on lifestyles and leisure activities at baseline were collected through a structured questionnaire administered by a trained staff member from the county Centers for Disease Control and Prevention. In the Chinese context there are some differences from western countries. For instance, in the Chinese context, leisure may involve playing Qigong and socializing by playing mahjong. Diet includes more rice than is typical in the west, though the diet is also quite varied with a variety of vegetables and meats. The questions used in the CLHLS survey were specifically relevant to the Chinese context and we coded these variables accordingly. Smoking status was divided into current, former, and never based on survey questions about current and past smoking history. Alcohol consumption was dichotomized as "drinker" if the respondent reported drinking one time per month or more in the last year [29]. Dietary pattern focused on fresh fruit, green leafy vegetables, legumes and their derivative product (e.g., tofu), and fish. Survey questions about frequency of eating each of these foods categorized responses into five levels: almost every day, once per week at least, once per month at least, occasionally, rarely or never. A participant that consumed at least three kinds of these foods at least once per week was coded into the healthy dietary pattern [29]. Marital status was dichotomized as “in marriage” if a participant was currently married with spouse present and “not in marriage” if divorced, widowed, separated or single. Four types of leisure activities were categorized [30]. Physical activity was based on the question “Do you do exercise regularly at present, including jogging, playing ball, running or Qigong, etc.?” and responses were coded as yes or no. Mental activities included reading books, magazines or newspapers. Social activities encompassed playing cards or mahjong, or participating in social groups or an organization for older people. Productive activities included housekeeping, gardening or raising domestic animals/pets. The frequency of participation in the latter three activities was grouped into “at least once per week”, which was considered as participating, versus less frequent.

We constructed four risk profiles from these factors, which were named low risk, medium-low, medium-high and high risk. The low risk profile consisted of participants conforming to three components: a healthy lifestyle (never smoking and having healthy diet), being married with spouse present and engaging in at least one leisure activity. Those with two, one and none of these elements were grouped into medium-low, medium-high, and high risk profile, respectively [8].

### Potential confounders

Demographic characteristics included age, sex, and region of residence; socioeconomic status included educational attainment, primary lifetime occupation, and economic condition; health status included functional limitation and chronic diseases. Primary lifetime occupation was defined as the longest-held job and classified into white collar versus others. Economic condition was trichotomized as good, fair, and poor according to the survey question “Compared with other local people, how do you rate your economic position?” Functional limitation was defined as the need for assistance in one or more items from a list of activities of daily living including bathing, dressing, eating, indoor transferring, toileting, and continence [31]. Chronic diseases were recorded by self-reported doctor diagnosis including hypertension, diabetes mellitus, cardiovascular disease, stroke, respiratory disease, and cancer.

### Statistical analysis

To distinguish the extent to which modifiable factors affect cognitive health expectancy, we define three terms: absolute increase, relative increase and relative reduction in years of life without CI. Absolute increase in years of life without CI refers to years of life being gained with all of these years free of CI. Relative increase and relative reduction in life years without CI assess the change in life years free of CI when there are gains in years both with and without CI. Relative increase refers to an increase in the proportion of life lived free of CI. Relative reduction refers to a decrease in the proportion of life lived free of CI.

We estimated total, cognitive impairment-free, and cognitive impaired life expectancy using a multistate life table approach [32]. This approach is based on two estimation steps. In the first step, the probability of transitions across states of health were estimated. The transitions were from (1) free of CI to CI (incidence of CI), (2) free of CI to death (mortality among those without CI), and (3) CI to death (mortality among those with CI). It was assumed transition from CI to free of CI was not possible, and therefore any observed transitions were treated as recording error [19]. An alternative was a model for misclassification, but there were too few cases transitioning from CI to free of CI (the proportion less than 2%) to stably include this model [33]. Due to the oversampling of those aged 80 and older, weights were applied to make the population age-sex-residence distribution equivalent to the 2000 census. Transition intensities were estimated using a Cox proportional hazards regression model assuming the instantaneous rate of transition was constant across observed time intervals. Since the precise date of onset of CI was not available, we utilized a continuous-time Markov process wherein participants were assumed to be able to develop CI at any time between observation points. Missing cognitive states were treated as censored. Interval censoring was used to consider individuals with missing states between two known cognitive states, and right censoring was used when an individual’s last state was missing, but they were known to still be alive.

In the second step, the transition probabilities were used as input into multistate life table functions as outlined by van den Hout and Matthews to derive estimates of life expectancy with and without CI [34]. 95% Confidence interval for differences in expectancies across categories of modifiable factors were calculated using bootstrapping with 500 replicates.

Due to known sex differences in life expectancy, analyses were performed separately for men and women. Models were first fitted separately for each modifiable factor and then for these factors aggregated into four risk profiles. All models were adjusted for demographic characteristics, socioeconomic factors, functional limitation, chronic diseases, and modifiable factors, if applicable.

Chronic diseases might be influenced by lifestyle behaviors, and in the causal pathway linking lifestyle behaviors with incident CI or death. To account for the potential mediating effect of chronic diseases, we repeated an analysis without adjusting for these chronic diseases. People in early stages of cognitive decline or near death might change their habits thus altering modifiable factors. To account for this possible reverse causation, we performed analyses excluding CI-free participants who experienced onset of CI during the first follow-up (n=1,209) and all participants who died in the first year (n=3,038) after their enrollment.

There was very little missing data for study variables (<2%). We applied multivariate imputation by chained equations generating five complete datasets to deal with missing values [35]. Variables included in the primary analyses were used in imputation models. Final statistical inferences were obtained by pooling estimates from imputed datasets according to Rubin’s rule [36]. Statistical analyses were implemented in R version 3.4.2 (R foundation for Statistical Computing).

## Supplementary Material

Supplementary Figure 1

Supplementary Tables
