# Supplementary material for: The association of healthy behaviors with cognitive health expectancy in Chinese older adults: a population-based cohort study
Source: Aging (Albany NY). 2020 Sep 9;12(17):16999–7021. doi: 10.18632/aging.103617 (PMC7521482; doi:10.18632/aging.103617)
Supplement: Supplementary Figure 1 [file aging-12-103617-s002..pdf]

SUPPLEMENTARY FIGURE

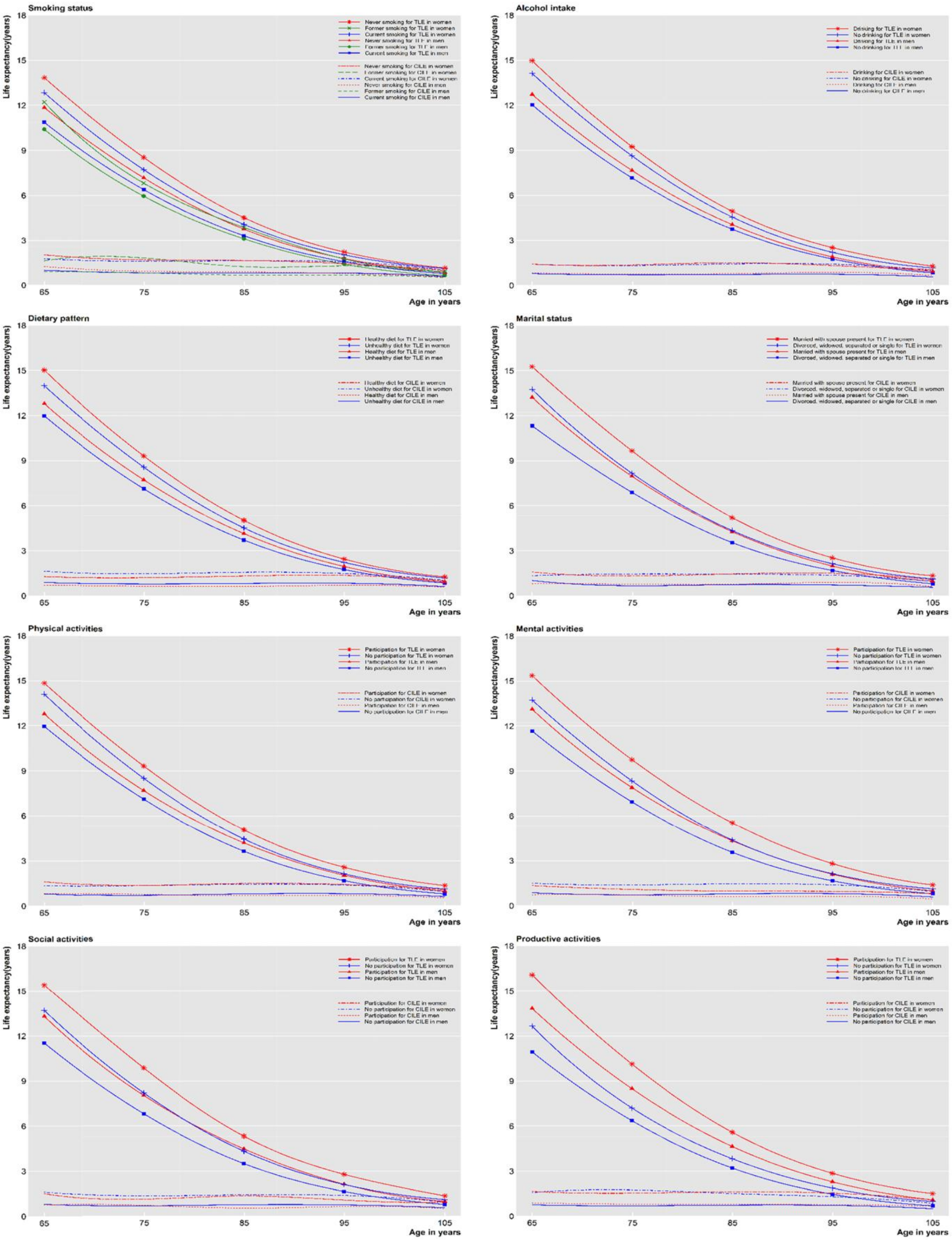

Supplementary Figure 1. Total life expectancy and cognitive impaired life expectancy for each modifiable factor after adjustment. TLE=Total life expectancy; CILE= Cognitive impaired life expectancy.
