# Supplementary material for: The association of healthy behaviors with cognitive health expectancy in Chinese older adults: a population-based cohort study
Source: Aging (Albany NY). 2020 Sep 9;12(17):16999–7021. doi: 10.18632/aging.103617 (PMC7521482; doi:10.18632/aging.103617)
Supplement: Supplementary Tables [file aging-12-103617-s001..pdf]

SUPPLEMENTARY TABLES

Supplementary Table 1. Differences in total life expectancy and cognitive impairment-free life expectancy, and proportion of life free of cognitive impairment by modifiable factors for men and women at age 65 years accounting for mediating effect.

Variables[a]	TLE	CIFLE	Proportion of CIFLE (%)	Difference in TLE (95%CI)	Difference in CIFLE (95%CI)	Difference in proportion of CIFLE (95%CI)
**Men**						
Smoking status						
Current	12.8	11.4	89.0	Reference	Reference	Reference
Never	13.9	12.2	87.6	1.0 (0.5 to 1.5)	0.7 (0.2 to 1.1)	-1.4 (-2.7 to -0.4)
Alcohol intake						
No drinking	14.1	13.0	92.0	Reference	Reference	Reference
Drinking	14.9	13.7	92.0	0.8 (0.3 to 1.2)	0.7 (0.3 to 1.2)	0 (-0.9 to 0.9)
Dietary pattern						
Unhealthy	14.1	12.8	91.0	Reference	Reference	Reference
Healthy	15.0	13.9	93.0	0.9 (0.3 to 1.3)	1.1 (0.5 to 1.5)	2.0 (1.4 to 4.9)
Marital status						
Not in marriage	13.4	12.0	89.3	Reference	Reference	Reference
In marriage	15.4	14.3	92.5	2.0 (0.3 to 2.6)	2.3 (0.8 to 2.8)	3.2 (2.1 to 15.1)
Physical activities						
No	14.1	12.9	91.7	Reference	Reference	Reference
Yes	15.0	13.8	92.1	0.9 (0.2 to 1.3)	0.9 (0.3 to 1.2)	0.4 (-0.2 to 3.4)
Mental activities						
No	13.7	12.5	90.9	Reference	Reference	Reference
Yes	15.3	14.3	93.0	1.6 (0.5 to 2.2)	1.8 (0.6 to 2.3)	2.1 (1.2 to 6.0)
Social activities						
No	13.6	12.4	91.4	Reference	Reference	Reference
Yes	15.5	14.4	92.8	1.9 (1.0 to 2.5)	2.0 (1.2 to 2.5)	1.4 (0.5 to 2.9)
Productive activities						
No	12.9	11.8	91.4	Reference	Reference	Reference
Yes	16.2	14.9	91.9	3.3 (0.9 to 3.8)	3.1 (1.0 to 3.6)	0.5 (-0.1 to 4.7)
**Women**						
Smoking status						
Current	15.0	12.5	83.9	Reference	Reference	Reference
Never	16.0	13.2	82.6	1.0 (0.5 to 1.5)	0.7 (0.2 to 1.1)	-1.3 (-2.9 to 0)
Alcohol intake						
No drinking	16.3	14.3	87.8	Reference	Reference	Reference
Drinking	17.3	15.3	88.4	1.0 (0.4 to 1.4)	0.9 (0.5 to 1.4)	0.6 (-0.6 to 2.3)
Dietary pattern						
Unhealthy	16.2	13.9	86.0	Reference	Reference	Reference
Healthy	17.3	15.5	89.5	1.1 (0.2 to 1.5)	1.6 (0.7 to 2.0)	3.5 (2.5 to 7.9)
Marital status						
Not in marriage	16.0	14.1	88.1	Reference	Reference	Reference

In marriage	17.5	15.4	87.5	1.6 (0.8 to 2.0)	1.3 (0.6 to 1.7)	-0.6 (-3.2 to 0.7)
Physical activities						
No	16.3	14.4	88.2	Reference	Reference	Reference
Yes	17.1	14.9	86.8	0.8 (0.3 to 1.2)	0.5 (0.1 to 0.8)	-1.4 (-3.9 to -0.5)
Mental activities						
No	15.9	13.8	86.7	Reference	Reference	Reference
Yes	17.7	15.8	89.3	1.8 (0.6 to 2.4)	2.0 (0.8 to 2.5)	2.6 (1.4 to 6.1)
Social activities						
No	15.8	13.8	87.3	Reference	Reference	Reference
Yes	17.7	15.6	88.1	1.9 (1.2 to 2.4)	1.8 (1.2 to 2.3)	0.8 (-0.5 to 2.0)
Productive activities						
No	14.7	12.5	85.3	Reference	Reference	Reference
Yes	18.5	16.2	87.4	3.8 (1.0 to 4.3)	3.7 (1.3 to 4.2)	2.1 (1.2 to 11.4)

TLE= total life expectancy; CIFLE= cognitive impairment-free life expectancy.

[a] Adjusted for age, region of residence, educational attainment, primary lifetime occupation, economic condition, functional limitation, and variables included in the table.

Supplementary Table 2. Differences in total life expectancy and cognitive impairment-free life expectancy, and proportion of life free of cognitive impairment by modifiable factors for men and women at age 85 years accounting for mediating effect.

Variables[a]	TLE	CIFLE	Proportion of CIFLE (%)	Difference in TLE (95%CI)	Difference in CIFLE (95%CI)	Difference in proportion of CIFLE (95%CI)
**Men**						
Smoking status						
Current	4.2	3.1	72.8	Reference	Reference	Reference
Never	4.7	3.5	73.7	0.5 (0.3 to 0.7)	0.4 (0.2 to 0.6)	0.9 (-1.6 to 2.9)
Alcohol intake						
No drinking	4.8	3.7	78.5	Reference	Reference	Reference
Drinking	5.1	4.0	78.5	0.4 (0.1 to 0.6)	0.3 (0.1 to 0.5)	0 (-1.6 to 1.6)
Dietary pattern						
Unhealthy	4.7	3.6	75.7	Reference	Reference	Reference
Healthy	5.2	4.4	83.0	0.5 (-0.2 to 0.8)	0.8 (0.2 to 1.0)	7.3 (5.5 to 15.7)
Marital status						
Not in marriage	4.5	3.5	76.8	Reference	Reference	Reference
In marriage	5.4	4.3	79.9	0.9 (0.1 to 1.1)	0.8 (0.2 to 1.1)	3.1 (1.3 to 8.6)
Physical activities						
No	4.6	3.5	76.3	Reference	Reference	Reference
Yes	5.3	4.3	82.0	0.7 (-0.2 to 1.0)	0.8 (0.1 to 1.0)	5.7 (3.6 to 16.1)
Mental activities						
No	4.6	3.4	75.6	Reference	Reference	Reference
Yes	5.5	4.6	84.2	0.9 (-0.1 to 1.3)	1.2 (0.2 to 1.5)	8.6 (6.2 to 23.3)
Social activities						
No	4.5	3.4	75.7	Reference	Reference	Reference
Yes	5.6	4.8	85.5	1.2 (0.3 to 1.6)	1.4 (0.8 to 1.8)	9.8 (7.1 to 18.6)
Productive activities						
No	4.1	3.1	74.7	Reference	Reference	Reference
Yes	5.9	4.7	80.7	1.8 (0.1 to 2.1)	1.7 (0.3 to 2.0)	6.0 (3.9 to 17.0)
**Women**						
Smoking status						
Current	5.1	2.9	56.8	Reference	Reference	Reference
Never	5.6	3.4	60.2	0.5 (0.3 to 0.7)	0.5 (0.3 to 0.6)	3.4 (0.3 to 5.8)
Alcohol intake						
No drinking	5.7	3.7	65.7	Reference	Reference	Reference
Drinking	6.2	4.1	66.6	0.5 (0.2 to 0.7)	0.4 (0.1 to 0.5)	0.9 (-0.8 to 2.9)
Dietary pattern						
Unhealthy	5.7	3.5	62.4	Reference	Reference	Reference
Healthy	6.3	4.4	71.1	0.6 (-0.2 to 0.9)	0.9 (0.2 to 1.1)	8.7 (6.9 to 12.0)
Marital status						
Not in marriage	5.5	3.5	64.0	Reference	Reference	Reference
In marriage	6.5	4.5	69.2	1.0 (0.1 to 1.3)	1.0 (0.2 to 1.2)	5.2 (2.8 to 8.6)
Physical activities						

No	5.6	3.6	65.0	Reference	Reference	Reference
Yes	6.3	4.3	67.8	0.7 (0 to 1.0)	0.6 (0.1 to 0.8)	2.8 (1.2 to 5.7)
Mental activities						
No	5.5	3.5	63.5	Reference	Reference	Reference
Yes	6.9	5.5	79.7	1.3 (-0.6 to 1.9)	2.0 (0.4 to 2.4)	16.2 (12.8 to 34.1)
Social activities						
No	5.4	3.5	63.9	Reference	Reference	Reference
Yes	6.6	4.7	71.5	1.2 (0.5 to 1.5)	1.2 (0.7 to 1.6)	7.6 (4.9 to 11.8)
Productive activities						
No	4.8	2.8	57.7	Reference	Reference	Reference
Yes	7.0	4.7	67.8	2.1 (0.2 to 2.6)	1.9 (0.4 to 2.4)	10.1 (7.5 to 14.8)

TLE= total life expectancy; CIFLE= cognitive impairment-free life expectancy.

[a] Adjusted for age, region of residence, educational attainment, primary lifetime occupation, economic condition, functional limitation, and variables included in the table.

Supplementary Table 3. Differences in total life expectancy and cognitive impairment-free life expectancy, and proportion of life free of cognitive impairment by modifiable factors for men and women at age 65 years accounting for reverse causation.

Variables[a]	TLE	CIFLE	Proportion of CIFLE (%)	Difference in TLE (95%CI)	Difference in CIFLE (95%CI)	Difference in proportion of CIFLE (95%CI)
**Men**						
Smoking status						
Current	12.4	11.9	95.7	Reference	Reference	Reference
Never	13.5	12.7	94.5	1.1 (0.5 to 1.5)	0.9 (0.4 to 1.4)	-1.2 (-1.5 to -0.6)
Alcohol intake						
No drinking	12.7	12.2	96.2	Reference	Reference	Reference
Drinking	13.3	12.8	96.0	0.6 (0.1 to 1.0)	0.5 (-0.1 to 1.0)	-0.2 (-0.5 to 0.3)
Dietary pattern						
Unhealthy	12.5	11.9	95.1	Reference	Reference	Reference
Healthy	13.6	13.2	97.0	1.1 (0.5 to 1.5)	1.3 (0.6 to 1.6)	1.9 (0.8 to 2.4)
Marital status						
Not in marriage	12.0	11.3	94.6	Reference	Reference	Reference
In marriage	13.8	13.2	95.9	1.8 (1.0 to 2.3)	1.9 (1.2 to 2.3)	1.3 (0.5 to 3.4)
Physical activities						
No	12.6	12.1	95.8	Reference	Reference	Reference
Yes	13.4	12.9	96.2	0.7 (0.3 to 1.1)	0.8 (0.3 to 1.1)	0.4 (0 to 0.9)
Mental activities						
No	12.2	11.7	95.5	Reference	Reference	Reference
Yes	13.8	13.4	96.6	1.6 (0.7 to 2.1)	1.7 (0.8 to 2.2)	1.1 (0.3 to 1.9)
Social activities						
No	12.2	11.7	96.0	Reference	Reference	Reference
Yes	13.9	13.5	97.1	1.7 (1.0 to 2.2)	1.8 (1.3 to 2.1)	1.1 (0.5 to 2.0)
Productive activities						
No	11.5	11.1	96.1	Reference	Reference	Reference
Yes	14.5	13.8	95.4	3.0 (2.0 to 3.3)	2.7 (1.9 to 3.2)	-0.7 (-1.0 to 0.8)
**Women**						
Smoking status						
Current	13.9	12.9	93.1	Reference	Reference	Reference
Never	15.0	13.8	91.8	1.1 (0.6 to 1.6)	0.8 (0.5 to 1.4)	-1.3 (-1.7 to -0.4)
Alcohol intake						
No drinking	14.2	13.3	93.4	Reference	Reference	Reference
Drinking	14.9	14.0	93.6	0.7 (0.1 to 1.2)	0.7 (0.2 to 1.1)	0.2 (-0.4 to 1.1)
Dietary pattern						
Unhealthy	13.9	12.6	91.3	Reference	Reference	Reference
Healthy	15.2	14.4	94.9	1.3 (0.6 to 1.8)	1.8 (0.9 to 2.1)	3.6 (1.7 to 4.8)
Marital status						
Not in marriage	13.9	13.1	94.5	Reference	Reference	Reference
In marriage	15.2	13.9	91.8	1.3 (0.8 to 1.7)	0.8 (0.4 to 1.4)	-2.7 (-3.5 to -1.0)
Physical activities						
No	14.2	13.3	93.6	Reference	Reference	Reference

Yes	14.8	13.7	92.4	0.6 (0.1 to 0.9)	0.4 (0 to 0.7)	-1.2 (-1.8 to -0.4)
Mental activities						
No	13.7	12.8	92.8	Reference	Reference	Reference
Yes	15.4	14.5	94.1	1.6 (0.8 to 2.2)	1.7 (0.9 to 2.2)	1.3 (0.3 to 2.3)
Social activities						
No	13.7	12.8	93.5	Reference	Reference	Reference
Yes	15.3	14.2	92.9	1.6 (1.0 to 2.0)	1.4 (0.9 to 1.9)	-0.6 (-1.4 to 0.3)
Productive activities						
No	12.6	11.5	91.2	Reference	Reference	Reference
Yes	16.0	14.8	92.4	3.4 (2.2 to 3.9)	3.3 (2.5 to 3.9)	1.2 (0.2 to 4.9)

TLE= total life expectancy; CIFLE= cognitive impairment-free life expectancy.

[a] Adjusted for age, region of residence, educational attainment, primary lifetime occupation, economic condition, functional limitation, chronic diseases, and variables included in the table.

Supplementary Table 4. Differences in total life expectancy and cognitive impairment-free life expectancy, and proportion of life free of cognitive impairment by modifiable factors for men and women at age 85 years accounting for reverse causation.

Variables[a]	TLE	CIFLE	Proportion of CIFLE (%)	Difference in TLE (95%CI)	Difference in CIFLE (95%CI)	Difference in proportion of CIFLE (95%CI)
**Men**						
Smoking status						
Current	3.7	3.2	86.5	Reference	Reference	Reference
Never	4.1	3.5	84.4	0.4 (0.2 to 0.6)	0.3 (0.1 to 0.5)	-2.1 (-2.9 to -0.5)
Alcohol intake						
No drinking	3.8	3.2	86.0	Reference	Reference	Reference
Drinking	4.1	3.6	87.1	0.3 (0.1 to 0.5)	0.3 (0.1 to 0.5)	1.1 (-0.1 to 3.0)
Dietary pattern						
Unhealthy	3.7	3.1	83.3	Reference	Reference	Reference
Healthy	4.2	3.8	90.6	0.5 (0.2 to 0.8)	0.8 (0.3 to 1)	7.3 (3.4 to 11.6)
Marital status						
Not in marriage	3.6	3.1	86.2	Reference	Reference	Reference
In marriage	4.3	3.7	86.1	0.7 (0.3 to 0.8)	0.6 (0.3 to 0.8)	-0.1 (-1.1 to 2.8)
Physical activities						
No	3.7	3.1	84.1	Reference	Reference	Reference
Yes	4.2	3.8	89.8	0.5 (0.1 to 0.7)	0.7 (0.3 to 0.8)	5.7 (2.4 to 10.6)
Mental activities						
No	3.6	3.0	83.4	Reference	Reference	Reference
Yes	4.4	4.1	91.6	0.9 (0.2 to 1.2)	1.1 (0.5 to 1.4)	8.2 (3.2 to 16.3)
Social activities						
No	3.5	3.0	84.1	Reference	Reference	Reference
Yes	4.5	4.2	92.2	1.0 (0.3 to 1.4)	1.2 (0.5 to 1.5)	8.1 (3.4 to 18.4)
Productive activities						
No	3.2	2.6	82.7	Reference	Reference	Reference
Yes	4.7	4.1	87.7	1.5 (0.6 to 1.8)	1.5 (0.7 to 1.8)	5.0 (1.6 to 13.4)
**Women**						
Smoking status						
Current	4.1	2.9	69.5	Reference	Reference	Reference
Never	4.7	3.5	73.5	0.6 (0.4 to 0.8)	0.6 (0.4 to 0.8)	4.0 (1.8 to 6.5)
Alcohol intake						
No drinking	4.4	3.2	74.5	Reference	Reference	Reference
Drinking	4.7	3.4	73.1	0.3 (0.1 to 0.5)	0.2 (0 to 0.3)	-1.4 (-3.1 to 0.4)
Dietary pattern						
Unhealthy	4.3	3.0	69.4	Reference	Reference	Reference
Healthy	4.9	3.9	80.4	0.6 (0.1 to 0.9)	0.9 (0.4 to 1.2)	11.0 (6.2 to 15.0)
Marital status						
Not in marriage	4.1	3.0	73.8	Reference	Reference	Reference
In marriage	5.0	3.8	75.5	0.9 (0.4 to 1.0)	0.7 (0.4 to 0.9)	1.7 (-0.3 to 6.1)
Physical activities						
No	4.3	3.1	73.6	Reference	Reference	Reference

Yes	4.8	3.6	75.2	0.5 (0.2 to 0.6)	0.5 (0.2 to 0.6)	1.6 (0.2 to 3.2)
Mental activities						
No	4.2	3.0	71.7	Reference	Reference	Reference
Yes	5.3	4.7	88.1	1.1 (0.2 to 1.7)	1.7 (0.7 to 2.0)	16.4 (8.0 to 29.5)
Social activities						
No	4.1	3.0	72.2	Reference	Reference	Reference
Yes	5.2	4.2	81.9	1.0 (0.3 to 1.4)	1.2 (0.6 to 1.6)	9.7 (4.7 to 19.0)
Productive activities						
No	3.6	2.2	62.8	Reference	Reference	Reference
Yes	5.4	4.1	75.3	1.8 (0.7 to 2.2)	1.8 (0.9 to 2.2)	12.5 (6.2 to 22.4)

TLE= total life expectancy; CIFLE= cognitive impairment-free life expectancy.

[a] Adjusted for age, region of residence, educational attainment, primary lifetime occupation, economic condition, functional limitation, chronic diseases, and variables included in the table.

Supplementary Table 5. Differences in total life expectancy and cognitive impairment-free life expectancy, and proportion of life free of cognitive impairment by four risk profile groups for men and women at age 65 and 85 years accounting for mediating effect.

Age	Variables[a]	High risk profile	Medium-high risk profile	Medium-low risk profile	Low risk profile	Difference of Medium-high *vs* High (95%CI)	Difference of Medium-low *vs* High (95%CI)	Difference of low *vs* High (95%CI)
65y	Men							
	TLE	9.3	12.0	13.8	15.5	2.7 (2.2 to 3.2)	4.5 (4.0 to 5.0)	6.2 (5.3 to 7.2)
	CIFLE	8.3	10.5	12.2	13.9	2.1 (1.7 to 2.7)	3.8 (3.5 to 4.4)	5.6 (4.9 to 6.7)
	Proportion (%)	89.6	87.4	88.0	89.9	-2.2 (-3.2 to -1.1)	-1.6 (-2.8 to -0.5)	0.3 (-1.8 to 2.4)
	Women							
	TLE	11.3	14.1	16.1	17.8	2.9 (2.4 to 3.4)	4.8 (4.3 to 5.3)	6.6 (5.6 to 7.6)
	CIFLE	9.5	11.5	13.2	15.1	2.0 (1.6 to 2.6)	3.7 (3.4 to 4.4)	5.6 (5.0 to 6.8)
	Proportion (%)	83.7	81.2	81.9	84.4	-2.5 (-4.1 to -1.0)	-1.8 (-3.6 to -0.2)	0.7 (-2.3 to 3.7)
85y	Men							
	TLE	2.6	3.7	4.5	5.2	1.1 (0.9 to 1.3)	1.9 (1.7 to 2.1)	2.6 (2.2 to 3.1)
	CIFLE	2.0	2.7	3.4	4.1	0.8 (0.6 to 1.0)	1.4 (1.3 to 1.6)	2.2 (1.8 to 2.6)
	Proportion (%)	74.3	73.1	74.8	78.6	-1.2 (-3.2 to 0.8)	0.5 (-1.8 to 2.4)	4.3 (0.3 to 7.8)
	Women							
	TLE	3.4	4.6	5.5	6.2	1.3 (1.1 to 1.4)	2.1 (1.9 to 2.4)	2.8 (2.4 to 3.4)
	CIFLE	2.0	2.7	3.3	4.1	0.7 (0.5 to 0.8)	1.3 (1.1 to 1.5)	2.0 (1.7 to 2.4)
	Proportion (%)	60.2	58.5	60.7	65.4	-1.7 (-4.0 to 0.6)	0.5 (-2.5 to 2.8)	5.2 (0.2 to 9.8)

TLE= total life expectancy; CIFLE= cognitive-impairment-free life expectancy.
[a] Adjusted for age, region of residence, educational attainment, primary lifetime occupation, economic condition, and functional limitation.

Supplementary Table 6. Differences in total life expectancy and cognitive impairment-free life expectancy, and proportion of life free of cognitive impairment by four risk profile groups for men and women at age 65 and 85 years accounting for reverse causation.

Age	Variables[a]	High risk profile	Medium-high risk profile	Medium-low risk profile	Low risk profile	Difference of Medium-high *vs* High (95%CI)	Difference of Medium-low *vs* High (95%CI)	Difference of low *vs* High (95%CI)
65y	Men							
	TLE	9.2	11.8	13.4	15.1	2.7 (2.0 to 3.0)	4.3 (3.3 to 4.6)	5.9 (4.6 to 6.5)
	CIFLE	8.9	11.3	12.7	14.4	2.4 (1.9 to 2.9)	3.8 (3.1 to 4.3)	5.5 (4.4 to 6.2)
	Proportion (%)	97.2	95.5	94.8	95.4	-1.7 (-1.9 to -0.6)	-2.4 (-2.6 to -0.6)	-1.8 (-2.3 to -0.1)
	Women							
	TLE	10.8	13.5	15.2	17.0	2.7 (2.1 to 3.2)	4.4 (3.6 to 4.9)	6.2 (4.9 to 6.9)
	CIFLE	10.3	12.5	13.8	15.6	2.2 (1.9 to 3.0)	3.6 (3.1 to 4.5)	5.4 (4.6 to 6.4)
	Proportion (%)	95.1	92.2	91.1	92.1	-2.9 (-3.1 to -0.7)	-4.0 (-4.2 to -1.1)	-3.0 (-3.7 to -0.3)
85y	Men							
	TLE	2.3	3.3	4.0	4.8	1.1 (0.7 to 1.2)	1.8 (1.3 to 2.0)	2.5 (1.7 to 2.7)
	CIFLE	1.9	2.7	3.4	4.2	0.9 (0.6 to 1.0)	1.5 (1.2 to 1.7)	2.3 (1.8 to 2.6)
	Proportion (%)	82.7	82.2	85.7	86.8	-0.5 (-1.3 to 0.5)	2.9 (0.3 to 3.1)	4.1 (0.2 to 6.1)
	Women							
	TLE	2.8	3.9	4.7	5.5	1.1 (0.8 to 1.2)	1.9 (1.3 to 2.1)	2.7 (1.9 to 2.8)
	CIFLE	2.1	2.9	3.6	4.3	0.8 (0.6 to 1.0)	1.5 (1.2 to 1.7)	2.2 (1.7 to 2.4)
	Proportion (%)	75.9	74.9	76.2	78.2	-0.9 (-1.3 to 2.6)	0.3 (-0.6 to 4.4)	2.4 (0 to 8.2)

TLE= total life expectancy; CIFLE= cognitive-impairment-free life expectancy.
[a] Adjusted for age, region of residence, educational attainment, primary lifetime occupation, economic condition, functional limitation, and chronic diseases.
